# Disseminated Mycobacterium Tuberculosis and IgA Nephropathy

**DOI:** 10.1155/2022/3785713

**Published:** 2022-11-01

**Authors:** Gordon Audley, Bianca Davidson, Erika Jones, Brendon Price, Brian Rayner, Nicola Wearne, Zibya Barday

**Affiliations:** ^1^Division of General Medicine, Department of Medicine, Faculty of Health Sciences, University of Cape Town and Groote Schuur Hospital, Cape Town, South Africa; ^2^Division of Nephrology and Hypertension, Department of Medicine, Faculty of Health Sciences, University of Cape Town and Groote Schuur Hospital, Cape Town, South Africa; ^3^Division of Anatomical Pathology, Department of Pathology, Faculty of Health Sciences, University of Cape Town/National Health Laboratory Services, Groote Schuur Hospital, Cape Town, South Africa

## Abstract

*Mycobacterium tuberculosis* (MTB) is an under-recognised cause of genitourinary disease. IgA nephropathy (IgAN), a leading cause of glomerulonephritis worldwide, has been described as a rare consequence of disseminated MTB infection. In this case report, we present the first case of MTB associated IgAN in Africa. Finding IgAN on kidney biopsy in an MTB endemic area should prompt a thorough investigation for MTB to increase the chance of remission of IgAN and prevent inappropriate use of immunosuppression.

## 1. Introduction

After COVID -19, *Mycobacterium tuberculosis* (MTB) is the second leading infectious cause of mortality globally [1]. South Africa is one of eight countries that account for two-thirds of the MTB global burden [1]. Despite this high prevalence of MTB, genitourinary TB is an often under-recognised extrapulmonary site of infection [2].

IgA nephropathy (IgAN) is the leading cause of glomerulonephritis [GN) worldwide [3]. In most cases, the cause remains unclear, leading to the diagnosis of primary IgAN [4]. Secondary IgAN is most often associated with infections, immune disorders, and malignancy [5]. Finding and treating an identified cause remains central to the otherwise largely supportive management approach to the disorder [6]. In a small series of case reports, IgAN has been linked to MTB [4]. In this case report, we present a rare case of IgAN in the setting of disseminated MTB.

## 2. Case Presentation and Results

A 41-year-old male, previously well, presented with a 2-year history of progressively worsening constitutional symptoms (fever, weight loss, and night sweats) and a dry cough. Additionally, he noted progressively worsening chronic lower back pain without sensory or motor fallout and a painful nonhealing ulcer on his right hallux. During this period, he presented several times to his local primary health care clinic where he was tested twice for MTB with a sputum Gene Xpert (GXP) test, which were both negative. He received symptomatic treatment for his back pain with paracetamol, nonsteroidal anti-inflammatory drugs (NSAIDs), and antibacterial cream for the ulcer on his toe.

**Figure 1:**
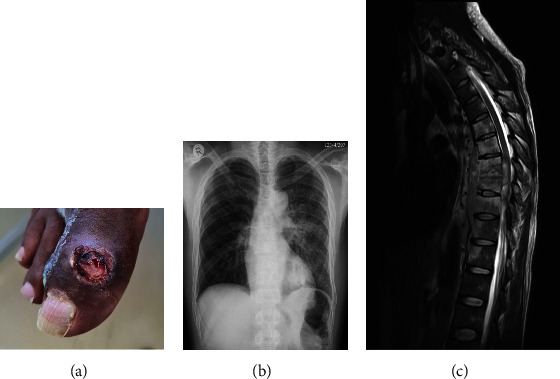
(a) Right hallux non-healing ulcer, (b) chest radiograph, and (c) magnetic resonance imaging (MRI) of the thoracic spine.

His first contact with our institute was via the vascular surgery outpatient clinic, where he was referred for the nonhealing ulcer on his right hallux (Figure 1(a)).  Table 1 reveals the blood investigations. The inflammatory markers were markedly elevated (erythrocyte sediment rate >130 mm/hr (normal range: less than 20 mm/hr) and C-reactive protein 113 mg/ml (normal range: less than 10 mg/ml)). Immunoglobulin A (IgA) levels were raised at 6.2 g/L (normal range: 0.7–4 g/L), measurement of circulating IgA-MTB antigen complexes was not available at our laboratory. There was a mildly raised anti-DNase B of 259 IU/ml (normal range: less than 200 IU/ml), with a negative autoimmune antibody panel (antinuclear antibodies, antidouble-stranded DNA antibody, and antineutrophil cytoplasmic antibodies). Testing for human immune virus (HIV), hepatitis B and C virus and syphilis were all negative. A nephrology consult was requested for a raised urine protein-creatinine ratio (uPCR) of 0.242 g/mmol creatinine.

A clinical examination found him to be normotensive, apyretic, and cachectic with no peripheral lymphadenopathy nor oedema. Tenderness upon palpation of his thoracic spine was noted with no associated neurological fallout. On dipsticks, he had 3+ leucocytes and 3+ red blood cells, while urine microscopy revealed granular casts with no bacterial culture growth.  Figure 1(b) shows a chest radiograph demonstrating bilateral reticular-nodular opacities.

An intensive MTB work up was performed. This included an induced sputum which tested trace positive for MTB on GXP and subsequently cultured MTB, sensitive to rifampicin and isoniazid. A biopsy of the right hallux ulcer (Figure 1(a)) revealed necrotising granulomatous inflammation with a solitary acid-fast bacillus seen on microscopy. Magnetic resonance imaging (MRI) of his spine (Figure 1(c)) revealed multi-level contiguous spondylitis at levels T6 to T10 with discitis and an epidural and paraspinal collection complicated by spinal cord compression but no oedema, in keeping with spinal MTB. No urine GXP nor urine TB cultures were sent.

The ultrasound showed bilateral normal-sized kidneys with increased echogenicity. A kidney biopsy was performed. The differential prior to kidney biopsy was acute interstitial nephritis (AIN) secondary to NSAIDs, direct infection of the kidney by MTB, or postinfectious glomerulonephritis.

**Figure 2:**
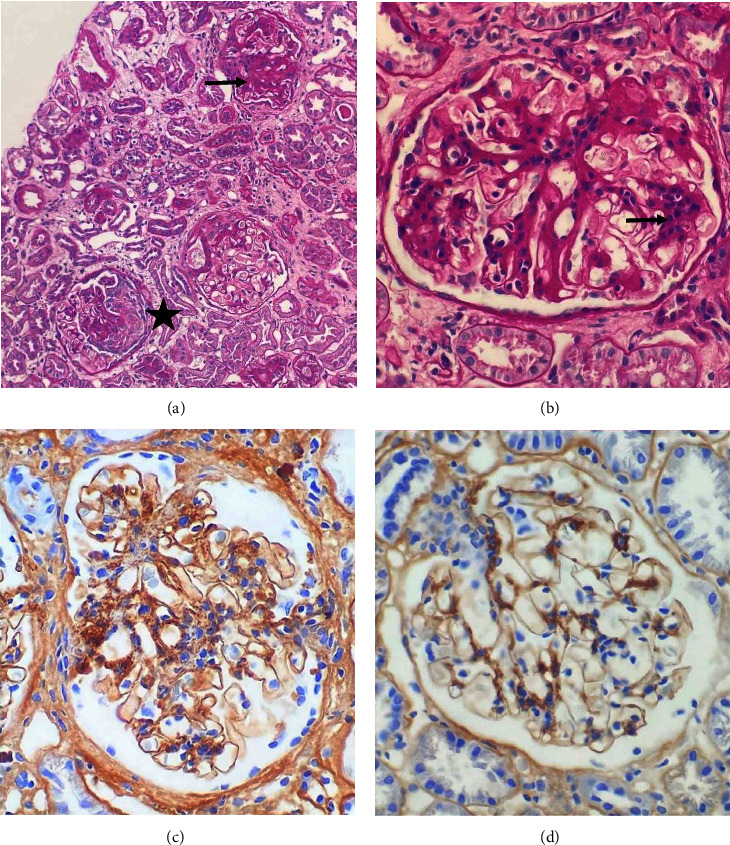
(a) Periodic acid-Schiff stained paraffin sections showing mesangial hypercellularity (arrow) and a focal cellular crescent (star) (x 100). (b) Higher magnification of glomerulus exhibiting mesangial hypercellularity (arrow) (x 400). (C) and (D) Immunohistochemical staining for IgA–strong mesangial and focal paramesangial staining, early subendothelial deposits are also appreciated (C). Strong mesangial staining was noted in occasional glomeruli which did not exhibit mesangial hypercellularity (D).

Kidney biopsy demonstrated a total of 59 glomeruli, four of which were globally sclerosed, three were segmentally sclerosed, and one showed a cellular crescent to be present. Haematoxylin and eosin (H&E) showed glomeruli with diffuse mesangial hypercellularity with no evidence of endocapillary hypercellularity (Figure 2(a) and 2(b)). The Jones methenamine silver stain revealed no double contours or spikes present, and the Congo Red did not show any evidence of amyloidosis. Immunohistochemical stains revealed diffuse strong IgA (3+) staining within the mesangium with lesser amounts noted within the paramesangium region (Figure 2(c) and 2(d)). Weak staining for IgM and C3 were noted (1+ intensity). IgG and C1q stains were negative. The background kidney showed mild tubular atrophy and interstitial fibrosis with a chronicity score of 2/10 (mild) as per Sethi et al. criteria [7,8]. No granulomata nor acid-fast bacilli were seen and no MTB were cultured. The diagnosis of IgAN (M_0_*E*_0_S_0_T_0_-C_1_) was assessed in the setting of disseminated MTB (pulmonary, spinal and skin) [9]. Early secreted antigenic target-6 staining was not available at our laboratory.

A multidisciplinary consultation (nephrology, orthopaedics, infectious diseases, and dermatology) advised first line MTB treatment with an extended course of 12 months (2 months intensive phase – (rifampicin/isoniazid, pyrazinamide/ethambutol), followed by 10 months of continuation phase (rifampicin/isoniazid)). An angiotensin-converting-enzyme inhibitor (enalapril) was prescribed for the proteinuria. At four months follow-up, the patient reported marked improvement in constitutional symptoms and back pain, while estimated GFR increased and uPCR also improved (Table 1).

## 3. Discussion

This report describes a rare case of secondary IgAN in the setting of MTB. To our knowledge, MTB-associated IgAN is limited to case reports and small case studies, with no cases described from Africa, despite the high prevalence and incidence of MTB throughout the continent. Kidney manifestations of MTB are diverse and heterogeneous, ranging from genitourinary TB, granulomatous interstitial nephritis, amyloidosis, treatment-related drug reactions, but rarely glomerulonephritis (GN). [2,10,11].

IgAN is the most prevalent primary cause of GN worldwide. The global prevalence rates vary according to ethnicity and geographical regions. It accounts for 40% of kidney biopsies in China and Japan, 30% in Europe, and 20% in the United States. [5] In Africa, IgAN appears to be comparatively less common and rare in the African black population [12]. In a 10-year retrospective analysis of native kidney biopsy reports in a tertiary centre in South Africa, IgAN was an infrequent cause of primary GN (5.8% of all identified cases) [13,14]. Furthermore, a meta-analysis of kidney biopsy findings across Africa between 1980 and 2004 found the prevalence of IgAN to be 2.8% [12]. The reasons for this are multifactorial and poorly understood.

The exact pathogenesis of MTB-IgAN is not well defined and an appropriate disease model is unavailable [4]. IgA, specifically against mycobacterial antigens, has been demonstrated in the sera of patients with active MTB, as have immune complexes of IgA antibodies and mycobacterial antigens [15]. The pathogenesis of IgAN is hypothesised to result from abnormally glycosylated IgA antibodies, which are mainly produced by mucosa-localized plasma cells in response to mucosal inflammation [4]. MTB antigens can stimulate *γδ*T cell activation and proliferation on the human mucosal surface to secrete a large amount of T cell-secreted transforming growth factor *β*1 (TGF*β*1) [4]. High TGF*β*1 levels stimulate B cells to differentiate into plasma cells [4]. These plasma cells subsequently produce defective IgA1 and promote IgA1 deposition in the mesangial areas of the kidney [4]. Deposition of these immune complexes, with subsequent activation of the alternative complement and the lectin pathway, with resultant local injury, leads to IgAN [15]. Identification in serum and demonstration on kidney biopsy of specific IgA-MTB antigen complexes and high TGF*β*1 levels, although not available in our laboratory, would strengthen the causal link between MTB and IgAN. Furthermore, why certain individuals develop this pathological IgA response to MTB is still unclear and requires further investigation.

When MTB infection affects the kidney or genitourinary tract, the diagnosis may be difficult. The conventional methods of diagnosis include: urine MTB culture, urine GXP, and urine lipoarabinomannan (LAM). Urine microscopy and mycobacterial culture remain the gold standards for diagnosis of genitourinary MTB; however, sensitivity is low, with a culture yield reported as around 46% in HIV-negative persons, and results take up to six weeks to become available [16]. Although data is limited, urine and renal tissue GXP have shown diagnostic promise in MTB-associated granulomatous interstitial nephritis (direct renal granulomatous infection) [17]. The utility of GXP on urine has been reported to have a sensitivity of 87% and specificity of 91% in a meta-analysis by Hillemann et al. in this setting [18]. Urine LAM is a valuable diagnostic tool in diagnosing renal involvement in disseminated MTB; however, its utility has only been demonstrated in human immune deficiency (HIV) positive patients with advanced immunosuppression and is thus not applicable in our case [19]. The role of urine MTB culture, GXP, and LAM in the setting of MTB associated IgAN (indirect immune mediated disease) has to our knowledge not been explored. Early secreted antigenic target-6 (ESAT-6), an antigen secreted by MTB, is implicated in the virulence and pathogenicity of MTB. Two case studies from China have demonstrated that ESAT-6 staining of the kidney tissue can assist in the diagnosis of MTB in the kidney [5,15]. Li et al. showed that when the culture is negative and ESAT-6 is positive, the chance of progressing to overt clinical MTB with immune suppression is high if no MTB prophylaxis is provided [15]. MTB treatment on the other hand, in the setting of IgAN and MTB, is associated with resolution of the proteinuria and haematuria [15].

In our resource-limited setting, without the advanced diagnostic techniques discussed above, we are unable to conclusively prove that our patient's disseminated MTB infection was indeed the cause of the IgAN; however, the rapid improvement in proteinuria as well as the clearance of haematuria with TB treatment strengthens our case of cause and effect.

## 4. Conclusion

In conclusion, in the setting where IgAN is a rare disease and MTB is common, the histological diagnosis of IgAN should prompt an extensive MTB workup. This will expedite the diagnosis and allow timely initiation of MTB treatment to increase the chance of remission of IgAN and prevent inappropriate use of immunosuppression with the increased risk of MTB dissemination [15].

## Figures and Tables

**Table 1:** Blood and urine investigation results.

	8 weeks prior to admission	7 weeks prior to admission	Admission	1^st^ week of admission	1 month follow-up	4 month follow-up
Sodium (136–145 mmol/l)	133	137	131	133	139	
Potassium (3.5–5.1 mmol/l)	5, 4	5, 4	5, 1	4, 6	4, 6	4.6
Urea (2.1–7.1 mmol/l)	6, 4	6, 2	4, 6	4, 3	2, 8	
Creatinine (64–104 umol/l)	90	102	94	87	67	70
Estimated GFR (ml/min/1.73 m^2^)	91	78	86	85	113	111
Calcium (2.20–2.55 mmol/l)	2, 15			2, 14	2, 32	
Magnesium (0.63–1.05 mmol/l)	0, 85			0, 71	0, 75	
Phosphate (0.78–1.42 mmol/l)	1, 12			1, 3	1, 21	
Total Protein (60–78 g/l)		76		71		
Albumin (35–52 g/l)	30	32		25	34	
White cell Count (3.90–12.60 × 10⁹/l)	7, 38	8, 34	9, 29	9, 76	5, 88	
Lymphocytes (1.40–4.50 (3.90–12.60 × 10⁹/l)		1, 29		1, 55		
Haemoglobin (13–17 g/dl)	12, 2	12, 1	11	9, 6	13, 1	
Platelets (186–454 × 10⁹/l)	587	646	710	603	529	
Total cholesterol (<5 mmol/l)	3, 33			3, 04		
Urine protein creatinine ratio (g/mmol creatinine)	0, 242		0, 137		0, 149	0.055

## Data Availability

No data set was needed.

## Additional Points

1. MTB is an important, albeit rare, secondary cause of IgAN. 2. Despite a high MTB prevalence in Africa–no cases of MTB associated IgAN have previously been described. 3. Finding IgAN in a MTB endemic area should prompt extensive work-up for MTB.
